# Laser Acupuncture Alleviates Symptoms and Improves Quality of Life in Women with Overactive Bladder: A Double-Blind, Pilot Randomized Controlled Trial

**DOI:** 10.1155/2020/1705964

**Published:** 2020-04-25

**Authors:** Yu-Wei Chang, Tsia-Shu Lo, Hsin-Ning Chang, Yi-Hsien Shiao, Yuan-Chieh Yeh

**Affiliations:** ^1^Department of Traditional Chinese Medicine, Chang Gung Memorial Hospital, Keelung Medical Center, Keelung, Taiwan; ^2^Division of Urogynecology, Department of Obstetrics and Gynecology, Chang Gung Memorial Hospital, Linkou Medical Center, Linkou, Taoyuan, Taiwan; ^3^Department of Obstetrics and Gynecology, Chang Gung Memorial Hospital, Keelung Medical Center, Keelung, Taiwan; ^4^Chang Gung University, School of Medicine, Taoyuan, Taiwan; ^5^Graduate Institute of Clinical Medicine Sciences, College of Medicine, Chang Gung University, Taoyuan, Taiwan; ^6^Program in Molecular Medicine, School of Life Sciences, National Yang Ming University, Taipei, Taiwan

## Abstract

**Objective:**

The aim of this study was to investigate the clinical efficacy of laser acupuncture for the treatment of women with overactive bladder (OAB) in Taiwan.

**Methods:**

A double-blind randomized controlled trial was conducted on female patients with OAB symptoms referred from gynecologists, and subjects were divided into two groups using blocked randomization. LaserPan (RJ-Laser, Germany) was applied to seven selected acupuncture points. The subjects received laser acupuncture 3 times per week for 3 weeks, 9 sessions in total. Basic patient data, Overactive Bladder Symptom Score (OABSS), Incontinence Impact Questionnaire (IIQ-7), and Urogenital Distress Inventory (UDI-6) scores were recorded prior to first treatment and at the end of 3^rd^, 6^th^, and 9^th^ treatments.

**Results:**

Thirty patients were enrolled, and twenty-seven patients completed all treatments in this study. The OABSS total score of the experimental group decreased significantly by 3.13 (*p* ≤ 0.001), 4.60 (*p* ≤ 0.001), and 3.79 (*p* ≤ 0.001) after 3^rd^, 6^th^, and 9^th^ treatments, respectively, compared with that of the control group. The IIQ-7 score declined significantly from baseline by 4.57 (*p*=0.003) and 3.63 (*p*=0.023) after 3^rd^ and 6^th^ treatments, respectively, compared with that of the control group. Similarly, the UDI-6 score of the experimental group exhibited a significant decrease from baseline by 1.90 (*p*=0.042) and 2.25 (*p*=0.025) after 6^th^ and 9^th^ interventions, respectively, compared with that of the control group.

**Conclusions:**

This study demonstrates that laser acupuncture can alleviate OAB symptoms and improve quality of life. This noninvasive device could be an effective therapy for women with OAB.

## 1. Introduction

According to International Continence Society, the overactive bladder (OAB) syndrome was defined in 2002 as “urgency, with or without urgency incontinence, usually with frequency and nocturia,” in the absence of proven infection or other obvious pathology [1]. In general, females suffered more from OAB syndrome than males in the world but the contributing factors remain to be elucidated [2]. According to statistics in different countries, the prevalence of OAB among females ranges from 6.0 to 17.4% [2, 3] and increases with age [4]. OAB has become a major women health issue worldwide. In Taiwan, the age-adjusted prevalence of OAB is 16% in men and 18.3% in women, respectively [5].

OAB poses significant financial impact in the US with medical costs ranging from USD 656 to 860 per patient annually [6]. In a US population-based survey of 2009, the total cost of disease-specific OAB was estimated at USD 24.9 to 36.5 billion [7]. Various methods for treating OAB have been developed. Conventional first-line interventions in the management of OAB include behavioral interventions such as fluid intake management, bladder training, and pelvic muscle exercises [8]. Oral antimuscarinics (such as solifenacin and tolterodine) or oral *β*3-adrenoceptor agonists (mirabegron) are commonly used as second-line treatments. However, dry mouth, constipation, vision irregularity, headache, hypertension, and other cardiovascular safety issues are of concern to patients treated with these drugs [9]. Clinicians may offer more invasive posterior tibial nerve stimulation (PTNS), intradetrusor onabotulinumtoxinA (Botox®) injection, and sacral neuromodulation (SNS) as third-line treatments for patients who are refractory to second-line therapies [10–12].

Traditional Chinese medicine (TCM) has for many years utilized herbal medicine, acupuncture, and moxibustion to treat lower urinary tract symptoms (LUTS) [13–15]. Some systematic reviews have concluded that acupuncture can improve OAB symptoms and enhance performance in objective urodynamic tests. However, both pain and hematoma limit the acceptance of acupuncture treatment [16, 17].

Laser acupuncture, belonging to the category of Low-Level Laser Therapy (LLLT), is commonly believed to be a safe and noninvasive treatment with no obvious side effect [18]. However, the therapeutic effect of laser acupuncture on women with OAB has not yet been examined. This study is a double-blind, pilot randomized controlled trial for assessing the efficacy of laser acupuncture on women with OAB in Taiwan.

## 2. Materials and Methods

### 2.1. Participants

This clinical trial was approved by the Institutional Review Board of Chang Gung Medical Foundation (Permit no. 201801679A3) and registered at ClinicalTrial.gov (ClinicalTrials.gov Identifier: NCT03829137).

According to prior research assessing the efficacy of moxibustion on patients with OAB [14], it was estimated that 14 people would be required for each arm to reach 90% statistical power. Whitehead et al. [19] also recommended pilot trial sample size per treatment arm of 15 for standardized effect size. Therefore, 30 participants were recruited for this clinical trial.

The recruited patients who met the inclusion criteria completed the urodynamic test (to rule out neurogenic bladder), per vaginal examination and initial screening (to rule out major surgery indication) by an experienced gynecologist. All participants started the trial after understanding thoroughly the study and signing informed consent forms.

The inclusion criteria were as follows:

1. Female at least 20 years old with differential diagnosis performed by a gynecologist prior to being referred to the Chinese Medicine Outpatient Clinic
2. Good health in general with no other major medical conditions and willing to sign the consent as well as responding to evaluation scales
3. Moderate to severe OAB symptoms with total scores of OABSS > 6 and the score of 3^rd^ question of OABSS > 2
4. Discontinuation of antimuscarinic and *β*3-adrenoceptor agonists for at least 2 weeks ensuring drug effect being washed out

The exclusion criteria were as follows:

1. Pregnancy or intention to get pregnant
2. Vaginal bleeding or urinary tract infection
3. Use of intradetrusor onabotulinumtoxinA injection (Botox®), PTNS, SNS, or other interventional therapies within 12 months prior to study enrollment
4. Other herbal and folklore therapies received to relieve OAB symptoms

### 2.2. Study Design

**Figure 1:**
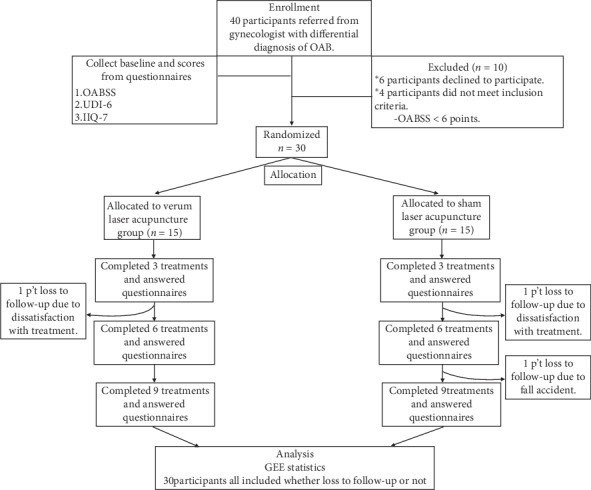
Flowchart of the study.

From early 2019, 40 patients were referred from the gynecology department and 30 patients met the inclusion criteria. The subjects were then divided using blocked randomization [20] into the experimental group receiving verum laser treatment (*n* = 15) and the control group receiving sham laser treatment (*n* = 15). All subjects receive laser acupuncture 3 times per week for 3 weeks, 9 sessions in total, with minor modifications according to clinical practice. Basic patient data, OABSS, UDI-6, and IIQ-7 scores were recorded prior to 1^st^ treatment and at the end of 3^rd^, 6^th^, and 9^th^ treatments. Adverse effects during the clinical trial were also recorded. The flowchart of this study is shown in Figure 1.

Neither patients nor investigators were informed of the grouping. Therefore, this study was a prospective double-blind randomized controlled trial.

### 2.3. Intervention

**Figure 2:**
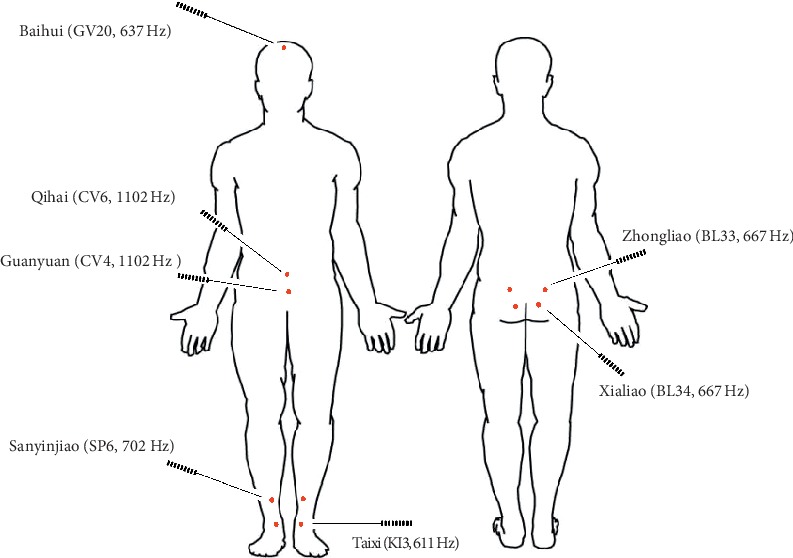
The seven selected acupuncture points and frequencies applied.

Acupuncture points including Taixi (KI3), Sanyinjiao (SP6), Guanyuan (CV4), Qihai (CV6), Zhongliao (BL33), Xialiao (BL34), and Baihui (GV20) were selected according to the WHO standardized acupressure point location guideline. The laser acupuncture treatment was administered by a licensed TCM physician. The patient laid in the supine position before treatment and the gallium aluminum arsenide LaserPan (maximal power, 150 mW; wavelength, 810 nm; area of probe, 0.13 cm^2^; power density, 1.19 W/cm^2^; pulsed wave; RJ-Laser, Reimers & Janssen GmbH, Waldkirch, Germany) was then applied to each selected acupoint. Subjects in the verum laser acupuncture group received a total energy of 6 joule at each acupoint with frequency prescribed according to Reininger Meridian Frequencies: Taixi (KI3, kidney; 611 Hz), Sanyinjiao (SP6, spleen; 702 Hz), Zhongliao (BL33, bladder; 667 Hz), and Xialiao (BL34, bladder; 667 Hz). However, the frequencies of Governor Vessel (GV) and Conception Vessel (CV) were undefined and frequencies of Guanyuan (CV4; Conception Vessel, 1102 Hz), Qihai (CV6; Conception Vessel, 1102 Hz), and Baihui (GV20; Governor Vessel, 637 Hz) were chosen according to experience (shown in Figure 2). Subjects in the control group received sham laser acupuncture treatment without laser output.

### 2.4. Outcome Measurements

Primary outcomes included Overactive Bladder Symptom Score (OABSS), whereas secondary outcomes included scores obtained using Incontinence Impact Questionnaire-Short Form (IIQ-7) and Urogenital Distress Inventory (UDI-6).

OABSS was developed for assessing symptom improvement and treatment efficacy in daily clinical practice [21]. OABSS comprising four symptom items (daytime frequency, nighttime frequency, urgency, and urgency incontinence), 15 points in total, translated into Chinese and validated in Taiwan [22, 23] was selected to be the primary outcome measurement in this study. Highly sensitive to treatment-related changes in OAB symptoms, OABSS can be an alternative to a bladder diary for symptom and efficacy assessment in daily clinical practice [21]. Both UDI-6 and IIQ-7 (Chinese version) which showed correlation with daytime urinary frequency and incontinent episodes [24] were employed to evaluate how OAB symptoms impact patients' quality of life (QoL).

### 2.5. Statistical Analysis

Analysis was performed using SPSS software (IBM Corp. Released 2015. IBM SPSS Statistics for Windows, Version 23.0. Armonk, NY: IBM Corp.). Values of *p* < 0.05 were considered to be statistically significant. Generalized estimating equation (GEE) was utilized to assess improvement ratios on scores of OABSS, UDI-6, and IIQ-7. Independent samples *T* test, Chi-square test, and nonparametric test (Mann-Whitney *U* test) were conducted to analyze baseline characteristics and raw data between the two groups.

## 3. Results

From early 2019, 40 patients referred from a gynecologist were screened and 30 were enrolled as participants. They were then randomly assigned to receive verum laser acupuncture (*n* = 15) or sham laser acupuncture (*n* = 15). Of them, 27 (90%) finished treatment; one patient in the experimental group dropped out because of schedule conflict and two patients in the control group discontinued the trial due to progressed OAB symptoms and hip fracture in a fall accident, respectively. The baseline characteristics were similar between the two groups (Table 1). A table of raw data from the three questionnaires showed the trend of score declining in time dependent manner between two groups calculated by nonparametric test (Table 2).

According to the logic calculus of GEE, data of the three subjects who did not complete the trial were still included in the statistics. The time point subtracted baseline intragroup and the slope between two groups were calculated using the generalized estimating equation (GEE) approach for the following results. *p* value < 0.05 represents the significant difference of the slopes between two groups.

### 3.1. Changes in OAB Symptoms

**Figure 3:**
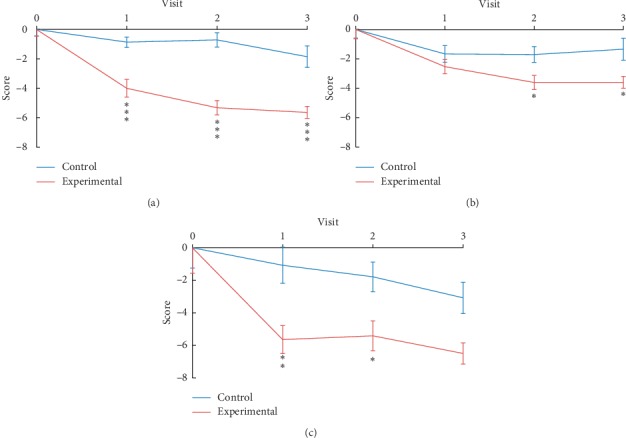
Line chart of primary and secondary outcomes. The mean change in OABSS (a), UDI-6 (b), and IIQ-7 (c) scores at 3^rd^, 6^th^, and 9^th^ treatments between experimental group and control group. Visits 1, 2, and 3 represent 3^rd^, 6^th^, and 9^th^ treatments, respectively. ^*∗*^*p* < 0.05, ^*∗∗*^*p* < 0.01, ^*∗∗∗*^*p* < 0.001.

In primary outcome measurement, the OABSS total score of the experimental group decreased significantly by 3.13 (*p* ≤ 0.001) after the 3^rd^ treatment compared with that of the control group. The difference in score of OABSS from baseline between the experimental group and control group changed was 4.60 (*p* ≤ 0.001) after the 6^th^ treatment and 3.79 (*p* ≤ 0.001) after the 9^th^ treatment (Table 3(a), Figure 3(a)).

### 3.2. Changes in QoL of Participants

After the 3^rd^ treatment, the UDI-6 score of the experimental group declined slightly (0.87), showing no significant change compared with that of the control group but exhibited a marked decrease from baseline after 6^th^ and 9^th^ interventions (−1.90; *p*=0.042 and −2.25; *p*=0.025, respectively) (Table 3(b)). For IIQ-7, the score of the experimental group exhibited a significant improvement from baseline after 3^rd^ and 6^th^ treatments (−4.57; *p*=0.003 and −3.62; *p*=0.023, respectively) compared with that of the control group, but only a slight insignificant decrease (−3.42; *p*=0.073) from baseline after the 9^th^ treatment (Table 3(c)). The GEE results are shown in Figures 3(b) and 3(c).

### 3.3. Daytime Urinary Frequency, Urgency, Nocturia, and Urgency Incontinence

**Figure 4:**
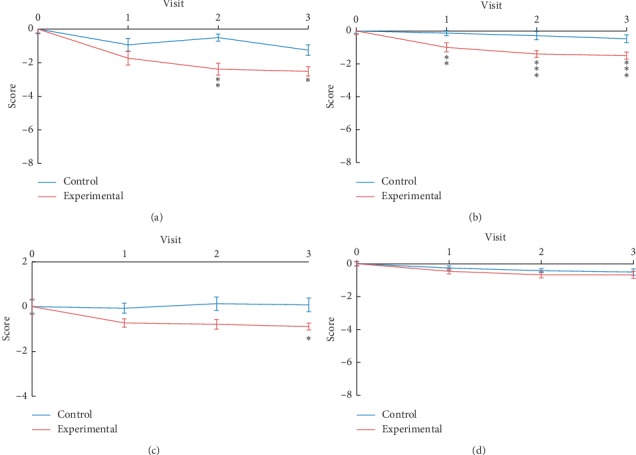
Line chart of the four questions of OABSS. The mean change urgency (Q3) (a), nocturia (Q2) (b), urgency incontinence (Q4) (c), and daytime urinary frequency (Q1) (d) between experimental group and control group. Visits 1, 2, and 3 represent 3^rd^, 6^th^, and 9^th^ treatments, respectively. ^*∗*^*p* < 0.05, ^*∗∗*^*p* < 0.01, ^*∗∗∗*^*p* < 0.001.

In view of urgency being the core OAB symptom, the question “how often do you have a sudden desire to urinate, which is difficult to defer” was taken from OABSS to assess specifically severity of urgency. The results showed the experimental group had marked decrease in urgency of statistical significance after 6^th^ and 9^th^ treatments (−1.88; *p*=0.001 and −1.26; *p*=0.030, respectively) compared with the control group (Table 4(a), Figure 4(a)). In addition, the symptoms of nocturia and urgency incontinence also improved significantly after laser acupuncture treatment (Tables 4(b) and 4(c); Figures 4(b) and 4(c)). There is no significant difference in daytime urinary frequency between the two groups (Table 4(d), Figure 4(d)).

## 4. Discussion

According to the American Urological Association (AUA) and the Society of Urodynamics, Female Pelvic Medicine and Urogenital Reconstruction (SUFU) OAB Guidelines, OAB therapy starts with dietary and behavioral modification and progresses to pharmacotherapy as well as third-line therapies including neuromodulation, PTNS, and so on. However, the guidelines highlight that each patient should not be required to go through each line of treatment in the order specified [8]. In view of the above-mentioned facts, many alternative treatments such as acupuncture, extracorporeal magnetic stimulation, Erbium: YAG laser treatment, elastomer roller perineal stimulation and, moxibustion have been developed and validated [14, 25–27]. To our knowledge, this is the first clinical trial assessing the therapeutic effect of laser acupuncture on patients with OAB.

In this study, patients with moderate to severe symptoms (OABSS > 6) and urgency more than once a week (Q3, 3^rd^ question of OABSS > 2) were enrolled, and the OABSS total scores were 8.7 and 8.5 (*p* = 0.76) in the experimental and control group, respectively, prior to treatment. After 3^rd^, 6^th^, and 9^th^ treatments, the OABSS total score decreased significantly in the experimental group in comparison with that in the control group, indicating that laser acupuncture can ameliorate moderate to severe OAB symptoms. Furthermore, the symptoms of urgency (Q3 of OABSS) and nocturia (Q2, 2^nd^ question of OABSS) improved significantly after 3^rd^, 6^th^, and 9^th^ treatments, with urgency incontinence (Q4, 4^th^ question of OABSS) improving after 9^th^ treatment, while daytime frequency (Q1, 1^st^ question of OABSS) showed an insignificant trend of decrease.

UDI-6 and IIQ-7 (Chinese version) are secondary outcome measurements in this study assessing the impact of OAB symptoms on patients' QoL. The IIQ-7 score improved significantly from baseline after 3^rd^ and 6^th^ treatments in the experimental group compared that in the control group, but the difference in improvement after the 9^th^ treatment between the two groups was insignificant (Table 3(c); Figure 3(c)). Such results can be attributed to related placebo effect in long-term treatment. The UDI-6 scale focuses on how troubling OAB symptoms are, especially urinary incontinence. The present findings revealed significant improvement in UDI-6 score in the experimental group after 6^th^ and 9^th^ interventions (Table 3(b); Figure 3(b)), which was consistent with the results obtained for Q4 of OABSS.

Acupuncture has been applied as OAB treatment for years. Previous research on OAB in females revealed that acupuncture could even be comparable to anticholinergics (tolterodine tartrate) in reducing the frequency of daytime urinary, incontinence, and nocturia and increase volume voided per micturition [28]. Moreover, other reviews suggested that acupuncture could reduce the frequency of urination, incontinence, and nocturia and increase the volume of urination [16, 17]. In view of related adverse effects such as pain, hematoma, and granuloma [16, 17], noninvasive laser acupuncture treatment may be a better option than needle acupuncture.

Laser acupuncture, a kind of LLLT has been used for more than 40 years in treating veterinary or human disease [29]. Being noninvasive and with minimal adverse effects, laser acupuncture has been promoted as a safer pain-free alternative to needle acupuncture. However, the mechanism, effectiveness, and indication of laser acupuncture are still unknown [18]. Until now, it remains a challenge to select appropriate parameters including wavelength, power output, energy dosage, and frequency in a specific situation due to heterogeneity among therapeutic theories and individuals' skin pigment, thickness, and location on the body [30]. In this study, selected acupoints were assumed to correspond to meridians, and the frequencies thus applied followed the Reininger Meridian Frequencies. The seven selected acupuncture points (KI3, SP6, CV4, CV6, GV20, BL33, and BL34) are commonly used in treating OAB, urinary incontinence, and LUTS according to TCM theory and the therapeutic effects have been demonstrated in many modern studies [16, 17]. We took these clinical trial papers and reviews as reference for selecting acupuncture points. Due to the characteristics of patient's posture changeable and short treatment time in laser acupuncture, the seven selected acupuncture points located in different parts of body can be stimulated in the same session. The reasons for selecting these acupuncture points concluded in Table 5 and the results of our clinical trial showed that these acupuncture points applied with Reininger Meridian Frequencies provide an effective treatment for women with OAB.

**Figure 5:**
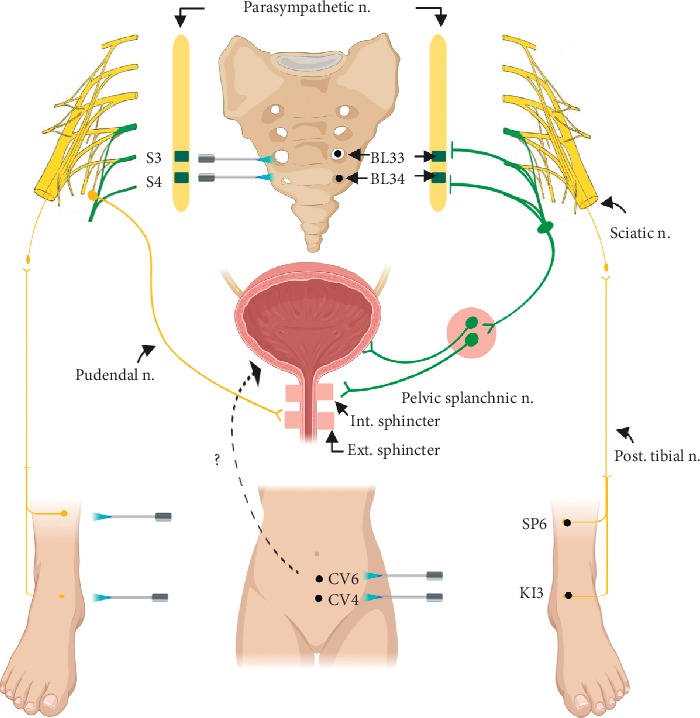
Speculative mechanism of laser acupuncture from neurological point of view.

To explain the possible mechanism from the neurological point of view, BL33 and BL34 are, respectively, located in the 3^rd^ and 4^th^ sacral foramen at the lumbosacral region, and sacral spinal nerves S2–S4 together form the pudendal nerve which innervates external urethral sphincter. SNS is an invasive therapeutic technique with electrodes placed in the S3–S4 sacral foramen to produce long-term electric stimulation of the sacral nerves for restoring normal voiding habits. The most well-accepted hypothesized mechanism for such effect is stimulation of the alpha myelinated afferent fibers and unmyelinated C fibers in the S3 and S4 pelvic, pelvic splanchnic nerve roots, and pudendal nerve roots that affect the micturition reflex [10, 35]. In our opinion, sacral neuromodulation theory may explain the possible mechanism of the stimulation of BL33 and BL34. Moreover, clinically available neuromodulation therapies for OAB target not only the sacral nerve roots but also the posterior tibial nerve and dorsal genital nerve for stimulation [12]. PTNS delivers retrograde stimulation to the sacral complex through electrodes placed near the posterior tibial nerve at locations similar to acupuncture points KI3 and SP6. However, the mechanism of laser acupuncture may be different from electrical stimulation provided by PTNS. As a consequence, we suppose the mechanism of these two acupuncture points may share some common pathways that needle acupuncture, laser acupuncture, and PTNS can reach. In addition, several studies used moxibustion on Conception Vessel meridian 3, 4, or 6 (CV3, CV4, or CV6) to alleviate OAB and other LUTS [14]. The speculative mechanism of moxibustion on CV meridian involves direct or indirect regulation of the autonomic nervous system (ANS) [36] but the exact principle behind is still unknown. Whether laser acupuncture achieves therapeutic effect through similar neurological mechanism or other theories, such as Qi or meridian theories, remains to be further studied. The speculative mechanism of laser acupuncture is shown in Figure 5.

### 4.1. Limitations

There are several validated questionnaires for OAB assessment being developed to evaluate the validity and reliability [23, 37]. However, these subjective assessment scales lack objective data. In recent years, many studies have reported that the nerve growth factor (NGF), brain-derived neurotrophic factor (BDNF), C-reactive protein (CRP), prostaglandins, and cytokines may be used as potential biomarkers of OAB but none of these meets the criteria for becoming an isolated OAB marker [38]. A potential biomarker, such as NGF, may be selected to be one of the secondary outcomes in future research.

Unlike traditional questionnaire with the same minimum and maximum score, OABSS assesses different items using different maximal scores (2, 3, 5, and 5 for daytime frequency, nighttime frequency, urgency, and urgency incontinence, respectively.). However, the frequency score of OABSS, ranging from 0 to 2, is too narrow to reflect the real situation of daytime urination frequency. For future research, it should be calculated using the number of urinations recorded in the 3-day bladder diary.

Constrained by limited research funding and resources, this study demonstrated only the short-term effect of laser acupuncture without a long-term follow-up. A long-term and multiple-center clinical trial is warranted for further evaluation.

## 5. Conclusion

To date, this is the first clinical trial evaluating the effects of laser acupuncture on OAB in women. The present results demonstrate that laser acupuncture can alleviate OAB symptoms and improve QoL of female suffering from OAB. Laser acupuncture may be a safe and effective intervention for treating OAB symptoms.

## Figures and Tables

**Table 1:** Baseline characteristics of participants.

Characteristics	Experimental group (*n* = 15)	Control group (*n* = 15)	*P* value
Age, mean (SD)	56.4 (14.3)	60.9 (10.2)	0.33^a^
Height (SD)	156.9 (4.2)	153 (6.2)	0.06^a^
Weight (SD)	59.5 (13.9)	60.83 (17.9)	0.90^c^
BMI (SD)	24.1 (4.6)	25.9 (7.3)	0.49^c^
Diabetes mellitus (%)	2 (13.3)	3 (20)	0.63^b^
HRT, *n* (%)	1 (6.7)	0 (0)	0.31^b^
Education, *n* (%)			0.30^b^
Junior high school or below	5 (33.3)	5 (33.3)	
Senior high school	6 (40.0)	9 (60.0)	
College or above	4 (26.7)	1 (6.7)	

Menopausal status, *n* (%)			0.65^b^
Pre	5 (33.3)	4 (26.7)	
Post	10 (66.7)	11 (73.3)	

Parity, mean (SD, range)	2.4 (1.4, 0–5)	2.3 (1.4, 0–5)	0.90^a^
Child delivery, *n* (%)			1.0^b^
Normal spontaneous delivery	11 (73.3)	11 (73.3)	
Cesarean section	2 (13.3)	2 (13.3)	

Gynecology-related surgery (%)	5 (33.3)	8 (53.3)	0.27^b^
Type of OAB (%)			0.69^b^
Wet OAB	10 (66.7)	11 (73.3)	
Dry OAB	5 (33.3)	4 (26.7)	

Urodynamic test, mean (SD)			
Urethral pressure profile: bladder capacity (ml)	292.3 (129.3)	262.6 (101.4)	0.51^c^
CMG + EMG: Pves-Pabd (CmH_2_O)	12.3 (28.5)	10.4 (21.2)	0.81^c^

Primary outcome, mean (SD)			
OABSS	8.7 (1.8)	8.5 (1.8)	0.74^c^
Q1 (daytime frequency)	1.5 (0.5)	1.7 (0.5)	0.22^c^
Q2 (nocturia)	2.3 (0.9)	2.4 (0.9)	0.81^c^
Q3 (urgency)	3.9 (0.9)	3.7 (1.1)	0.78^c^
Q4 (urgency incontinence)	1.1 (1.4)	0.7 (1.2)	0.33^c^

Secondary outcome, mean (SD)			
UDI-6	6.0 (2.5)	5.7 (2.4)	0.62^c^
IIQ-7	9.9 (5.7)	10.8 (3.9)	0.41^c^

^a^Independent samples *T* test. ^b^Chi-square test, Pearson Chi-square, asymptotic significance (2-sided). ^c^Nonparametric test (Mann-Whitney *U* test). SD: standard deviation. BMI: body mass index. HRT: hormone replacement therapy. CMG: cystometry. EMG: external sphincter electromyogram. Pves: vesical pressure. Pabd: abdominal pressure. OABSS: overactive bladder symptom score. Q1: 1^st^ question of OABSS, Q2: 2^nd^ question of OABSS, Q3: 3^rd^ question of OABSS, Q4: 4^th^ question of OABSS. UDI-6: Urogenital Distress Inventory. IIQ-7: Incontinence Impact Questionnaire.

**Table 2:** Raw data of OABSS, UDI-6, and IIQ-7.

Outcome	Experimental (*n*)	Control (*n*)	*p*
OABSS (mean ± SD)			
Baseline	8.7 ± 1.8 (15)	8.5 ± 1.8 (15)	0.74
Visit 1	4.7 ± 2.4 (15)	7.7 ± 1.4 (15)	≤0.001^*∗∗∗*^
Visit 2	3.4 ± 1.9 (14)	7.8 ± 1.9 (14)	≤0.001^*∗∗∗*^
Visit 3	3.1 ± 1.6 (14)	6.7 ± 2.8 (13)	≤0.001^*∗∗∗*^

UDI-6 (mean ± SD)			
Baseline	6.0 ± 2.5 (15)	5.7 ± 2.4 (15)	0.62
Visit 1	3.5 ± 1.9 (15)	4.1 ± 2.3 (15)	0.41
Visit 2	2.3 ± 1.8 (14)	3.9 ± 2.1 (14)	0.03^*∗*^
Visit 3	2.4 ± 1.6 (14)	4.3 ± 2.9 (13)	0.03^*∗*^

IIQ-7 (mean ± SD)			
Baseline	9.9 ± 5.7 (15)	10.8 ± 3.9 (15)	0.41
Visit 1	4.2 ± 2.3 (15)	9.9 ± 4.2 (15)	≤0.001^*∗∗∗*^
Visit 2	4.2 ± 3.2 (14)	9.4 ± 3.9 (14)	0.001^*∗∗*^
Visit 3	3.0 ± 2.5 (14)	8.5 ± 3.7 (13)	≤0.001^*∗∗∗*^

OABSS: overactive bladder symptom score. IIQ-7: Incontinence Impact Questionnaire. UDI-6: Urogenital Distress Inventory. SD: standard deviation. ^*∗*^*p* < 0.05, ^*∗∗*^*p* < 0.01, ^*∗∗∗*^*p* < 0.001, calculated using nonparametric test (Mann–Whitney *U* test).

**Table 3:** Generalized estimating equation analysis of outcome measurements. OABSS (a) as primary outcome, UDI-6 (b), and IIQ-7 (c) as secondary outcomes. (*N* = 30). ^*∗*^*p* < 0.05, ^*∗∗*^*p* < 0.01, ^*∗∗∗*^*p* < 0.001.

	*ß*	SE	*P*
*(a)*			
Group			
Experimental	0.20		0.753
Control	0^a^		
Group *∗* time course (experimental vs. control)			
3^rd^ treatment vs. baseline	−3.13	0.87	≤0.001^*∗∗∗*^
6^th^ treatment vs. baseline	−4.60	0.91	≤0.001^*∗∗∗*^
9^th^ treatment vs. baseline	−3.79	1.09	≤0.001^*∗∗∗*^

*(b)*			
Group			
Experimental	0.33		0.699
Control	0^a^		
Group *∗* time course (experimental vs. control)			
3^rd^ treatment vs. baseline	−0.87	0.99	0.382
6^th^ treatment vs. baseline	−1.90	0.93	0.042^*∗*^
9^th^ treatment vs. baseline	−2.25	1.01	0.025^*∗*^

*(c)*			
Group			
Experimental	−2.16		0.309
Control	0^a^		
Group *∗* time course (experimental vs. control)			
3^rd^ treatment vs. baseline	−4.57	1.51	0.003^*∗∗*^
6^th^ treatment vs. baseline	−3.63	1.60	0.023^*∗*^
9^th^ treatment vs. baseline	−3.42	1.91	0.073

a. Set to zero because this parameter is redundant. *ß*. Estimate parameter. SE: standard error.

**Table 4:** Generalized estimating equation analysis of the four questions of OABSS. Urgency in Q3 (a), nocturia in Q2 (b), urgency incontinence in Q4 (c), and daytime urinary frequency in Q1 (d). (*N* = 30) ^*∗*^*p* < 0.05, ^*∗∗*^*p* < 0.01, ^*∗∗∗*^*p* < 0.001.

	*β*	SE	*p*
*(a)*			
Group			
Experimental	−1.974*E* − 16		1.000
Control	0^a^		
Group *∗* time course (experimental vs. control)			
3^rd^ treatment vs. baseline	−0.80	0.65	0.220
6^th^ treatment vs. baseline	−1.88	0.57	0.001^*∗∗*^
9^th^ treatment vs. baseline	−1.26	0.58	0.030^*∗*^

*(b)*			
Group			
Experimental	−0.27		0.327
Control	0^a^		
Group *∗* time course (experimental vs. control)			
3^rd^ treatment vs. baseline	−0.87	0.31	0.005^*∗∗*^
6^th^ treatment vs. baseline	−1.11	0.31	≤0.001^*∗∗∗*^
9^th^ treatment vs. baseline	−1.02	0.29	≤0.001^*∗∗∗*^

(c)			
Group			
Experimental	0.47		0.297
Control	0^a^		
Group *∗* time course (experimental vs. control)			
3^rd^ treatment vs. baseline	−0.67	0.47	0.157
6^th^ treatment vs. baseline	−0.93	0.47	0.050
9^th^ treatment vs. baseline	−0.98	0.47	0.038^*∗*^

(d)			
Group			
Experimental	−0.342		0.053
Control	0^a^		
Group *∗* time course (experimental vs. control)			
3^rd^ treatment vs. baseline	−1.92	0.20	0.336
6^th^ treatment vs. baseline	−2.48	0.20	0.224
9^th^ treatment vs. baseline	−1.73	0.31	0.580

^a^Set to zero because this parameter is redundant. *ß*. Estimate parameter. SE: standard error.

**Table 5:** The reasons for the acupuncture points selection.

Acupoints (location)	The reasons for the acupuncture points selection	Possible mechanism	Reference
BL33 and BL34 (lumbar sacrum region)	The acupuncture points frequently used in LUTS are BL31 to BL35. They are located above the sacral foramen lie over the first to fourth sacral nerve roots, respectively. These acupuncture points correspond to the segmental innervation of bladder's parasympathetic nerve. A RCT published in JAMA confirmed the effect of electroacupuncture involving the lumbosacral region for women with SUI.	Sacral nerve stimulation.	[31]

KI3 and SP6 (lower leg)	According to TCM theory, OAB and LUTS are due to Zang-Fu organ imbalance of kidney, spleen, and liver. KI3 (kidney meridian) and SP6 (spleen meridian) are commonly used to adjust Yin-Yang balance in OAB and LUTS and have been included in several studies.	Posterior tibial nerve stimulation.	[28, 32]

CV4 and CV6 (lower abdomen)	In TCM theory, bladder dysfunction is due to insufficiency of Yang Qi. Conception Vessel meridian is suggested as a treatment option as several studies used moxibustion or acupuncture on CV3, CV4, or CV6 to alleviate OAB and other LUTS.	Autonomic nervous system regulation.	[14, 33]

GV20 (head)	GV20, one of the most important acupoints of the government vessel, is commonly used in neurology and psychiatry. Stimulation at GV20 will increase cerebral perfusion in the cerebral cortex, stabilize emotions, and reduce sympathetic activity in stress response. Studies have reported stimulating GV20 can lift fallen Yang Qi in accordance with TCM theory; therefore, we selected GV20 to calm emotions and lift patient's Yang Qi.	Increasing the cerebral perfusion in the cerebral cortex for mood stabilization.	[34]

LUTS: lower urinary tract symptoms. RCT: randomized controlled trial. JAMA: The Journal of the American Medical Association. SUI: Stress Urinary Incontinence. TCM: traditional Chinese medicine.

## Data Availability

The data supporting the findings of this study are available from the corresponding author upon request.

## References

[1] Wein A. J., Rovner E. S. (2002). Definition and epidemiology of overactive bladder. *Urology*.

[2] Temml C., Heidler S., Ponholzer A., Madersbacher S. (2005). Prevalence of the overactive bladder syndrome by applying the international continence society definition. *European Urology*.

[3] Wang Y., Xu K., Hu H. (2011). Prevalence, risk factors, and impact on health related quality of life of overactive bladder in China. *Neurourology and Urodynamics*.

[4] Gacci M., Sebastianelli A., Spatafora P. (2018). Best practice in the management of storage symptoms in male lower urinary tract symptoms: a review of the evidence base. *Therapeutic Advances in Urology*.

[5] Yu H. J., Liu C. Y., Lee K. L., Lee W. C., Chen T. H. (2006). Overactive bladder syndrome among community-dwelling adults in Taiwan: prevalence, correlates, perception, and treatment seeking. *Urologia Internationalis*.

[6] Powell L. C., Szabo S. M., Walker D., Gooch K. (2018). The economic burden of overactive bladder in the United States: a systematic literature review. *Neurourology and Urodynamics*.

[7] Onukwugha E., Zuckerman I. H., McNally D., Coyne K. S., Vats V., Mullins C. D. (2009). The total economic burden of overactive bladder in the United States: a disease-specific approach. *The American Journal of Managed Care*.

[8] Lightner D. J., Gomelsky A., Souter L., Vasavada S. P. (2019). Diagnosis and treatment of overactive bladder (Non-Neurogenic) in adults: AUA/SUFU guideline amendment 2019. *Journal of Urology*.

[9] Chapple C. R., Siddiqui E. (2017). Mirabegron for the treatment of overactive bladder: a review of efficacy, safety and tolerability with a focus on male, elderly and antimuscarinic poor-responder populations, and patients with OAB in Asia. *Expert Review of Clinical Pharmacology*.

[10] Sukhu T., Kennelly M. J., Kurpad R. (2016). Sacral neuromodulation in overactive bladder: a review and current perspectives. *Research and Reports in Urology*.

[11] de Wall L. L., Heesakkers J. P. (2017). Effectiveness of percutaneous tibial nerve stimulation in the treatment of overactive bladder syndrome. *Research and Reports in Urology*.

[12] Jaqua K., Powell C. R. (2017). Where are we headed with neuromodulation for overactive bladder?. *Current Urology Reports*.

[13] Wang Y., Zhishun L., Peng W., Zhao J., Liu B. (2013). Acupuncture for stress urinary incontinence in adults. *Cochrane Database of Systematic Reviews*.

[14] Lee H. Y., Yun Y. J., Choi J. Y. (2018). Effectiveness and safety of moxibustion for alleviating symptoms of overactive bladder: a prospective, randomized controlled, crossover-design, pilot study. *Medicine (Baltimore)*.

[15] Lee W.-C., Liu Y.-L. (2018). Traditional Chinese medicine and herbal supplements for treating overactive bladder. *Urological Science*.

[16] Forde J. C., Jaffe E., Stone B. V., Te A. E., Espinosa G., Chughtai B. (2016). The role of acupuncture in managing overactive bladder; a review of the literature. *International Urogynecology Journal*.

[17] Zhao Y., Zhou J., Mo Q., Wang Y., Yu J., Liu Z. (2018). Acupuncture for adults with overactive bladder: a systematic review and meta-analysis of randomized controlled trials. *Medicine (Baltimore)*.

[18] Chon T. Y., Mallory M. J., Yang J., Bublitz S. E., Do A., Dorsher P. T. (2019). Laser acupuncture: a concise review. *Medical Acupuncture*.

[19] Whitehead A. L., Julious S. A., Cooper C. L., Campbell M. J. (2016). Estimating the sample size for a pilot randomised trial to minimise the overall trial sample size for the external pilot and main trial for a continuous outcome variable. *Statistical Methods in Medical Research*.

[20] Efird J. (2010). Blocked randomization with randomly selected block sizes. *International Journal of Environmental Research and Public Health*.

[21] Homma Y., Kakizaki H., Yamaguchi O. (2011). Assessment of overactive bladder symptoms: comparison of 3-day bladder diary and the overactive bladder symptoms score. *Urology*.

[22] Hung M. J., Chou C. L., Yen T. W. (2013). Development and validation of the Chinese overactive bladder symptom score for assessing overactive bladder syndrome in a RESORT study. *Journal of the Formosan Medical Association*.

[23] Chou E. C., Hung M. J., Yen T. W. (2014). The translation and validation of Chinese overactive bladder symptom score for assessing overactive bladder syndrome and response to solifenacin treatment. *Journal of the Formosan Medical Association*.

[24] Chan S. S., Choy K. W., Lee B. P. (2010). Chinese validation of urogenital distress inventory and incontinence impact questionnaire short form. *International Urogynecology Journal*.

[25] Lo T. S., Tseng L. H., Lin Y. H., Liang C. C., Lu C. Y., Pue L. B. (2013). Effect of extracorporeal magnetic energy stimulation on bothersome lower urinary tract symptoms and quality of life in female patients with stress urinary incontinence and overactive bladder. *Journal of Obstetrics and Gynaecology Research*.

[26] Lin Y. H., Hsieh W. C., Huang L., Liang C. C. (2017). Effect of non-ablative laser treatment on overactive bladder symptoms, urinary incontinence and sexual function in women with urodynamic stress incontinence. *Taiwanese Journal of Obstetrics and Gynecology*.

[27] Iimura K., Watanabe N., Masunaga K. (2016). Effects of a gentle, self-administered stimulation of perineal skin for nocturia in elderly women: a randomized, placebo-controlled, double-blind crossover trial. *PLoS One*.

[28] Yuan Z., He C., Yan S., Huang D., Wang H., Tang W. (2015). Acupuncture for overactive bladder in female adult: a randomized controlled trial. *World Journal of Urology*.

[29] Jang I., Sun S., Jeong M. (2019). Early history of laser acupuncture: who used it first?. *Integrative Medicine Research*.

[30] Wang Y. (2016). Laser acupuncture and local laser therapy in veterinary medicine with overview of applied laser types and clinical uses. *AJTCVM*.

[31] Liu Z., Liu Y., Xu H. (2017). Effect of electroacupuncture on urinary leakage among women with stress urinary incontinence: a randomized clinical trial. *JAMA*.

[32] Aydogmus Y., Sunay M., Arslan H., Aydin A., Adiloglu A. K., Sahin H. (2014). Acupuncture versus solifenacin for treatment of overactive bladder and its correlation with urine nerve growth factor levels: a randomized, placebo-controlled clinical trial. *Urologia Internationalis*.

[33] Barnes M. A., Bennett J., Ross J., Kraemer K., Cotton S. (2018). Single-point Acupuncture for treatment of urge incontinence in women: a pilot nonrandomized trial. *Medical Acupuncture*.

[34] Litscher G., Wang L., Wang X., Gaischek I. (2013). Laser acupuncture: two acupoints (baihui, neiguan) and two modalities of laser (658 nm, 405 nm) induce different effects in neurovegetative parameters. *Evidence Based Complementary and Alternative Medicine*.

[35] Leng W. W., Chancellor M. B. (2005). How sacral nerve stimulation neuromodulation works. *Urologic Clinics North America*.

[36] Yun S. P., Jung W. S., Park S. U. (2007). Effects of moxibustion on the recovery of post-stroke urinary symptoms. *The American Journal of Chinese Medicine*.

[37] Groenendijk I. M., Scheepe J. R., Noordhoff T. C., Blok B. F. M. (2019). The validation of the Dutch OAB-q SF: an overactive bladder symptom bother and health-related quality of life short-form questionnaire. *Neurourology and Urodynamics*.

[38] Wrobel A. F., Kluz T., Surkont G. (2017). Novel biomarkers of overactive bladder syndrome. *Ginekologia Polska*.

